# A Chromosome-Scale Genome of *Trametes versicolor* and Transcriptome-Based Screening for Light-Induced Genes That Promote Triterpene Biosynthesis

**DOI:** 10.3390/jof11010081

**Published:** 2025-01-20

**Authors:** Yang Yang, Xuebo Hu

**Affiliations:** 1 Institute for Medicinal Plants, College of Plant Science and Technology, Huazhong Agricultural University, Wuhan 430070, China; life333@webmail.hzau.edu.cn; 2 Innovation Academy of International Traditional Chinese Medicinal Materials, Huazhong Agricultural University, Wuhan 430070, China

**Keywords:** *Trametes versicolor*, chromosome-level, phylogenetic evolution, CAZymes, CYP450, triterpene biosynthesis

## Abstract

*Trametes versicolor* is an important fungus with medicinal properties and a significant role in lignocellulose degradation. In this study, we constructed a high-quality chromosome-level genome of *T. versicolor* using Illumina, PacBio HiFi, and Hi-C sequencing technologies. The assembled genome is 47.42 Mb in size and contains 13,307 protein-coding genes. BUSCO analysis revealed genome and gene completeness results of 95.80% and 95.90%, respectively. Phylogenetic analysis showed that *T. versicolor* is most closely related to *T. pubescens*, followed by *T. cinnabarina* and *T. coccinea*. Comparative genomic analysis identified 266 syntenic blocks between *T. versicolor* and *Wolfiporia cocos*, indicating a conserved evolutionary pattern between the two species. Gene family analysis highlighted the expansion and contraction of genes in functional categories related to the biosynthesis of secondary metabolites, including several *T. versicolor*-specific genes. Key genes involved in lignocellulose degradation and triterpene production were identified within the CAZyme and CYP450 gene families. Transcriptomic analysis under dark and light conditions revealed significant changes in the expression of genes related to secondary metabolism, suggesting that light signals regulate metabolic pathways. A total of 2577 transporter proteins and 2582 membrane proteins were identified and mapped in the *T. versicolor* genome, and 33 secondary metabolite gene clusters were identified, including two light-sensitive triterpene biosynthesis clusters. This study offers a comprehensive genomic resource for further investigation into the functional genomics, metabolic regulation, and triterpene biosynthesis of *T. versicolor*, providing valuable insights into fungal evolution and biotechnological applications.

## 1. Introduction

*Trametes versicolor*, also known as *Coriolus versicolor* and *Polyporus versicolor*, is an important medicinal fungus widely distributed in nature, demonstrating significant research value in various fields such as medicine [1,2,3], ecology [4,5], and industry [6]. *T. versicolor* possesses remarkable immunomodulatory, antitumor, and anti-inflammatory effects, as demonstrated in multiple studies. Its extracts, including polysaccharide-K (PSK) and polysaccharide-peptide (PSP), have been widely used as adjuncts in cancer treatment [7,8]. Rich in bioactive polysaccharides, dietary fiber, high-quality proteins, trace elements, and low-calorie compounds, *T. versicolor* holds substantial nutritional and health benefits [9]. As a white-rot fungus, it efficiently decomposes lignocellulose, particularly lignin, cellulose, and hemicellulose, thus playing a pivotal role in the carbon cycle of forest ecosystems [10]. In recent years, with the rise of multi-omics technologies, significant progress has been made in research on *T. versicolor*’s ecological functions, medicinal components, and biodegradation. Despite its vast potential in medicine, food, and environmental applications, the molecular genetic mechanisms and genomic features of *T. versicolor* remain insufficiently explored. A deeper understanding of its genome is crucial for uncovering its complex metabolic product synthesis and environmental adaptability, which are of significant scientific importance for further research.

*T. versicolor*, a white-rot fungus, belongs to the Polyporaceae family within Basidiomycota [11]. It is widely distributed across the globe, particularly in the primeval forests of the northern hemisphere [12]. In China, *T. versicolor* is commonly found throughout the southeast, southwest, and northwest regions, where it primarily parasitizes broadleaf trees and decaying wood at altitudes above 3000 m [13]. The morphological characteristics of *T. versicolor* exhibit noticeable regional variations, particularly in terms of cap color, size, and surface structure. The cap of *T. versicolor* displays concentric bands of various colors, typically including brown, yellow, white, and red. These color differences are often closely linked to environmental factors such as temperature and humidity. In tropical regions, the cap colors are more vibrant, while in colder regions, they tend to be more muted. The flesh is usually thin and leathery, and the spores are oval shaped with a smooth surface. There may be some variation in the size and shape of the spores among different populations of *T. versicolor* from different regions. Although morphological studies laid the foundation for the early classification of *T. versicolor*, the limitations of this approach have led researchers to shift towards more precise molecular biology techniques. While molecular biology has made significant progress in the classification of many fungi, research on the molecular biology of *T. versicolor* remains relatively limited. Traditional methods, such as morphological observation, physiological characterization, and the analysis of ribosomal RNA gene regions [14], have notable limitations in uncovering genetic differences among *T. versicolor* populations. Morphological and physiological traits are often influenced by environmental factors, which may obscure the underlying genetic differences and result in low resolution. Similarly, ribosomal RNA gene regions, although widely utilized, frequently lack enough polymorphisms to distinguish closely related populations or strains. Moreover, these traditional approaches are typically restricted to specific loci, limiting their ability to capture genome-wide genetic diversity and structural variations. In recent years, the rapid advancements in genomics and high-throughput sequencing technologies have enabled an increasing number of studies to leverage whole-genome data, offering deeper insights into the classification, genetic relationships, and evolutionary history of *T. versicolor*.

Carbohydrate-active enzymes (CAZymes) and cytochrome P450 (CYP450) enzymes are two critical gene families in fungi, playing central roles in wood degradation and secondary metabolite synthesis [15,16]. CAZymes degrade complex polysaccharides into simpler sugars and oxidized compounds through a variety of hydrolases and oxidoreductases, providing essential carbon sources for fungal growth. The CAZymes includes subfamilies such as glycoside hydrolases (GHs), glycosyltransferases (GTs), lignocellulose-degrading monooxygenases (LPMOs), and auxiliary activity enzymes (AAs). In white-rot fungi, CAZymes are the genetic foundation for lignocellulose degradation, which is crucial for carbon cycling and the development of bioenergy from biomass [17]. Cytochrome P450 enzymes (CYP450s) are a class of monooxygenases containing heme cofactors, playing a crucial role in the secondary metabolic pathways of fungi [18]. For example, studies have shown that CYP450 genes in the genus *Aspergillus* are involved in the synthesis of aflatoxins, and regulating the expression of these enzymes can influence the synthesis and accumulation of toxins [19]. In medicinal fungi, CYP450 enzymes are primarily involved in the synthesis and modification of bioactive compounds, such as triterpenoids. The genetic diversity of these enzymes contributes to the complexity of fungal metabolic products [20]. The quantity and functional distribution of these two gene families in fungi directly influence their ecological roles and metabolic potentials, making them key areas of research. However, systematic studies on CAZymes and CYP450 in *T. versicolor* are still lacking, and further exploration is needed to understand their functional differentiation, tissue-specific expression, and evolutionary patterns.

Light is a critical environmental factor influencing fungal growth, development, and metabolism, including the regulation of key enzymes involved in lignocellulose degradation and secondary metabolite biosynthesis [21,22]. Among these enzymes, CAZymes play a central role in breaking down complex plant cell wall polysaccharides into simple sugars, which serve as essential nutrients for fungal development and precursors for secondary metabolic pathways. Under light induction, CAZymes contribute to enhanced polysaccharide formation in *Lentinula edodes*, with key gene families involved, such as cellulases (GH6, GH7, GH45, AA9), hemicellulases (CE1, CE3, GH10, GH11), pectinases (GH28, PL1, CE8), and ligninases (AA1, AA3) [23]. Transcriptomic studies in *Pleurotus eryngii* have identified 319 differentially expressed CAZyme genes during the transition from primordium to fruiting body formation under light [24], while in *L. edodes*, the expressions of 6 GHs, 4 AAs, 3 CEs, and 3 GTs were significantly upregulated [25]. These studies suggest that light may induce CAZyme expression by activating transcriptional pathways involved in lignocellulosic degradation, enabling fungi to adapt to their environment and enhancing secondary metabolite production. However, the molecular mechanism of photoregulation of CAZyme and its specific role in fungal development are still lacking. Research has shown that light influences not only fungal morphology but also the synthesis of specific secondary metabolites, such as pigments, antibiotics, and triterpenoids [26]. Triterpenoids, as key secondary metabolites in many fungi, exhibit significant pharmacological activities, including anticancer and anti-inflammatory effects, and their synthesis is strongly regulated by light signals [27,28]. Light enhances the expression of key enzymes in the triterpenoid biosynthesis pathway, such as HMG-CoA reductase and CYP450, thereby significantly increasing triterpenoid production [20]. Additionally, light signals activate transcription factors, such as Zn2Cys6-type transcription factors, which, in turn, enhance the activity of secondary metabolite gene clusters and promote the synthesis of these metabolites [29]. In *T. versicolor*, triterpenoids are the main active components responsible for antitumor, anti-inflammatory, and immunomodulatory activities [27]. However, research on the biosynthesis pathways of specific triterpenoid products, such as coriolus acid or coriolus ketone, and their molecular mechanisms of light regulation remains limited. This lack of understanding hinders further exploration into the directed production of high-value triterpenoid compounds.

Here, we successfully constructed a high-quality, chromosome-level genome of *T. versicolor* by integrating Illumina, PacBio, and Hi-C sequencing technologies. We further characterized the genome and established its phylogenetic position within the fungal kingdom. Comparative genomic analysis was conducted to identify and functionally compare the CYP450 and CAZyme families. Transcriptomic analysis revealed how light regulates *T. versicolor*’s metabolism and gene expression. This comprehensive genomic analysis provides valuable molecular data for future systematic studies of *T. versicolor*.

## 2. Materials and Methods

### 2.1. Sampling Information

The obtained fungal cultures were inoculated onto 20 glucose potato dextrose agar (PDA) plates coated with glass paper and incubated in the dark at 28 °C for 10 days. The mycelia were then collected, frozen in liquid nitrogen, and used for genome sequencing and chromosome-level genome assembly. Fungal genomic DNA was extracted using the CTAB method [30], and the integrity and purity of the extracted DNA samples were assessed using 1% agarose gel electrophoresis and quantified with a NanoDrop 2000 spectrophotometer (Thermo Scientific, Wilmington, DE, USA). The DNA concentration was precisely quantified using a Qubit 3.0 fluorometer (Thermo Scientific, Waltham, MA, USA).

### 2.2. Genome Size Assessment

The genome size of *T. versicolor* was evaluated using the K-mer counting method. Jellyfish (v2.3.1) [31] was used to calculate the K-mer frequency distribution in cleaned sequencing data, selecting the appropriate K-mer size. GenomeScope 2.0 [32] was used to evaluate genome size, heterozygosity, and repeat content. The size estimate of the genome was obtained by combining the peak K-mer frequency and GenomeScope analysis.

### 2.3. Genome Sequencing and Assembly

A high-quality whole genome sequence was obtained using a combined approach with both Illumina and PacBio platforms. Short-read libraries (300 bp insert size) were constructed on the Illumina HiSeq X platform (Illumina Inc., San Diego, CA, USA), while long-read libraries (20 kb insert size) were constructed on the PacBio Revio platform (Pacific Biosciences, Menlo Park, CA, USA), followed by sequencing. For chromosome-level genome assembly, Hi-C sequencing was performed on the Illumina HiSeq X platform in paired-end mode (PE150), generating high-quality Hi-C data.

PacBio HiFi sequences from the *T. versicolor* genome were assembled using Hifiasm (v0.19.9) [33] with default settings. The initial assembly was then polished with Illumina short reads using NextPolish (v1.4.1) [34], resulting in a complete contig-level assembly. Clean reads from Hi-C sequencing were aligned to the genome using HapHiC (v1.0.3) (https://github.com/zengxiaofei/HapHiC, accessed on 22 October 2024) [35]. Manual corrections to the anchoring were made using Juicebox (v1.13) [36], resulting in the final chromosome-level genome assembly. The integrity of the genome was assessed using BUSCO (v5.1.2) [37], comparing it to the fungal lineage dataset fungi_odb10.

### 2.4. Gene Prediction and Annotation

To identify repetitive sequences in the *T. versicolor* genome, an integrated approach combining de novo prediction and known repeat libraries was employed. We used a customized pipeline based on the RepeatModeler (v2.0.5) [38] tool to perform de novo repetitive sequence identification. Repetitive sequences identified by RepeatModeler were then masked in the genome using RepeatMasker (v4.0.9) [39], which was run with the constructed repeat library. For greater accuracy, the RepBase database was incorporated into the repeat annotation pipeline to identify known repeat elements not captured during the de novo prediction.

Gene prediction was conducted using a combination of ab initio, homology-based, and RNA-Seq-based methods to ensure comprehensive annotation of the *T. versicolor* genome. Gene models were initially predicted using the Augustus (v3.4) [40] tool, which is optimized for de novo gene prediction in fungal genomes. Additionally, SNAP (v2.0) [41] was applied as a complementary tool to generate gene models based on machine learning algorithms trained on fungal genome datasets. To enhance the accuracy of the ab initio predictions, homologous gene prediction was carried out using the GeMoMa (v1.9) [42], which utilized sequence alignment to fungal genomes to refine and validate the predicted gene models. The predicted gene models from the different approaches were integrated into a non-redundant gene set using EVidenceModeler (v1.1) [43].

Functional annotation of the predicted gene models was carried out by comparing the sequences against several publicly available protein and nucleotide databases, including the NCBI NR database, UniProt, and InterPro. Gene Ontology (GO) terms were assigned to each gene model to classify their molecular functions, biological processes, and cellular components. Additionally, KEGG (Kyoto Encyclopedia of Genes and Genomes) pathway analysis was conducted to predict the metabolic pathways involved, providing insights into the potential biochemical roles of identified genes. snRNA prediction was performed using Infernal (v1.1.2) [44], with alignment to the Rfam database for the identification of rRNAs and microRNAs. tRNA models were predicted using tRNAscan (v2.0.9) [45], while rRNA identification was carried out using BLASTn (v2.7.1).

### 2.5. Gene Family Clustering and Phylogenetic Analysis

To explore the evolutionary features of gene families in the *T. versicolor* genome, genomic sequences from 29 fungal species (including *T. versicolor*, *Antrodiella citrinella*, *Daedalea quercina*, *Dichomitus squalens*, *Fibroporia radiculosa*, *Fomitopsis pinicola*, *F. rosea*, *Ganoderma sinense*, *G. lucidum*, *Gelatoporia subvermispora*, *Grifola frondosa*, *Laetiporus sulphureus*, *Lentinula edodes*, *Lentinus tigrinus*, *Obba rivulosa*, *Phanerochaete carnosa*, *P. chrysosporium*, *Phlebia centrifuga*, *Phlebiopsis gigantea*, *Polyporus arcularius*, *P. brumalis*, *Poria cocos*, *Postia placenta*, *Sparassis crispa*, *Steccherinum ochraceum*, *Trametes cinnabarina*, *T. coccinea*, *T. pubescens*, and *Wolfiporia cocos*) were retrieved from the Ensembl database. OrthoFinder (v2.5.4) [46] was used for orthologous gene clustering and gene family identification based on protein sequences. Single-copy genes identified from the clusters were aligned using MAFFT (v7.471) [47]. The optimal substitution model was automatically selected using ModelFinder, and a phylogenetic tree was constructed with IQ-TREE (v2.0.6) [48], applying the maximum likelihood method and 1000 bootstrap replicates. Divergence times were sourced from the TimeTree database (http://www.timetree.org/, accessed on 26 October 2024), and species divergence was estimated using the mcmctree package in PAML (v4.10.7) [49]. Gene family expansions and contractions were detected based on the phylogenetic tree using CAFÉ (v4.2.1) [50]. GO and KEGG enrichment analyses for the expanded and contracted gene families were performed using the R package clusterProfiler.

### 2.6. Comparative Genomic Analysis

Gene synteny analysis was conducted using MCScanX (v1.0) [51]. To identify syntenic regions across species, BLASTp (v2.7.1) (E < 1 × 10^−5^) was employed for both all-vs.-all and self-comparisons. The resulting synteny data were visualized with JCVI.

### 2.7. Identification Members of CAZymes, CYP450, and Transport Proteins

The gene families of carbohydrate-active enzymes (CAZymes) and cytochrome P450s (P450s) in *T. versicolor* were identified using the genome annotation and sequence files. For CAZymes, HMMER 3.0 was used to search for conserved protein domains and motifs associated with CAZyme families. Hidden Markov Model (HMM) profiles specific to CAZyme families were downloaded from the CAZy database (http://www.cazy.org, accessed on 29 October 2024). These profiles represent characteristic domains and conserved motifs of key enzyme families, including glycoside hydrolases (GHs), glycosyltransferases (GTs), carbohydrate esterases (CEs), polysaccharide lyases (PLs), and auxiliary activities (Aas). Protein sequences obtained from the *T. versicolor* genome annotation were used as query input for HMMER. The hmmscan module was employed to compare these sequences against the CAZy HMM profiles. Matches with an E-value ≤ 1 × 10^−5^ were considered statistically significant, indicating potential CAZyme candidates. Proteins matching specific HMM profiles were assigned to their corresponding CAZyme families. To ensure accuracy, redundant hits and non-specific matches were manually inspected and filtered based on domain architecture and functional annotation. Cytochrome P450 genes were identified by retrieving the P450 domain models (Pfam ID: PF00067) from the Pfam database (http://pfam.xfam.org/, accessed on 5 November 2024) and using HMMER 3.0 to search the *T. versicolor* protein sequences with a stringent E-value threshold (E < 1 × 10^−5^). Additionally, protein sequences of CAZymes and P450s from other fungi were downloaded from the NCBI database and subjected to local BLASTp comparisons against the *T. versicolor* genome using the same E-value cutoff (≤10^−5^). The results from both approaches were merged. The transporter database was downloaded from the Transporter Classification Database (https://www.tcdb.org/, accessed on 8 November 2024), and a BLASTp search was performed with an e-value threshold set to 10^−5^.

### 2.8. Transcriptome Analysis

The mycelium of *T. versicolor*, collected during its active growth phase, was exposed to two conditions: complete darkness (control group) and normal light (treatment group) for 5 days. For each condition, three biological replicates were prepared. These biological replicates were derived from independent starter cultures grown under identical conditions to ensure reproducibility. Each biological replicate was further divided into three technical replicates, with mycelia inoculated onto separate plates. The plates within each biological replicate were prepared from the same starter culture, but all biological replicates originated from different cultures to capture biological variability. After treatment, the mycelium was quickly frozen in liquid nitrogen and stored at −80 °C for RNA extraction. The RNA was extracted following a previously established method [52], and a cDNA library was subsequently constructed. These libraries were sequenced on the Illumina HiSeq X platform. Following sequencing, raw data were processed with FastQC for quality control, and adapter sequences and low-quality reads were removed using fastp (v0.23.4) [53]. Cleaned data were aligned to the *T. versicolor* genome using Hisat2 (v2.2.1) [54] with default parameters. Aligned reads were assembled with StringTie (v2.2.1) [55], and gene expression was quantified by calculating FPKM values using Cufflinks (v2.2.1) [56]. Differentially expressed genes were identified with a fold change (FC) ≥ 2 or ≤0.5 and a *p*< 0.05.

### 2.9. qRT-PCR Analysis

To validate the reliability of the transcriptome sequencing data, ten genes were randomly selected for verification (*FUN_011163*, *FUN_004807*, *FUN_002029*, *FUN_004597*, *FUN_008142*, *FUN_006342*, *FUN_004598*, *FUN_006074*, *FUN_000932*, and *FUN_002993*). RNA from the different treatment groups was reverse transcribed to obtain first-strand cDNA. Gene-specific primers were designed using Primer Premier 5.0, with Actin as the internal control (Table S1). Gene expression levels were calculated using quantitative PCR (qPCR) based on the 2^−ΔCt^ method. The ΔCt value was determined by subtracting the cycle threshold (Ct) of the target gene from that of the reference gene. The reference gene used for normalization was *GAPDH*, which was selected based on their stable expression across the experimental conditions. For fold-change analysis between experimental groups, the 2^−ΔΔCt^ method was applied, where ΔΔCt represents the difference between the ΔCt values of the experimental and control groups. The data were statistically analyzed using a *t*-test, with significance set at *p* < 0.05, and the error bars represent the standard deviation (SD).

## 3. Results

### 3.1. Genome Sequencing and Assembly

**Figure 1:**
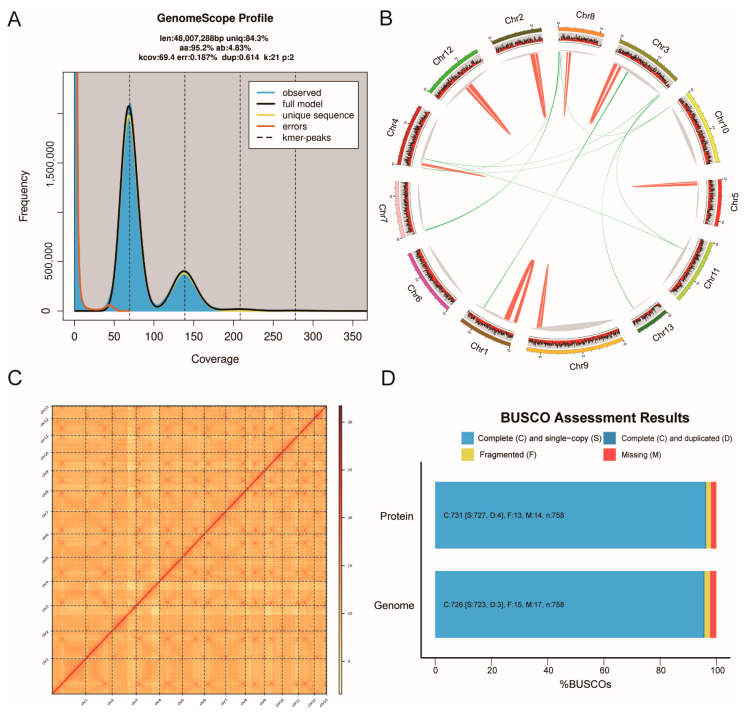
(**A**) Genome size estimation of *T. versicolor* based on K-mer frequency analysis. The histogram represents the distribution of K-mer frequencies, with the major peak indicating the estimated genome size. (**B**) The genome assembly circular map of *T. versicolor* displays the chromosome lengths and coding regions as the outermost colored blocks. The red heat map in the center indicates gene density across each window. The innermost lines represent the collinearity within and between chromosomes. The green line in the circle indicates genes that are collinear with the genome of *T. versicolor*, and the red lines represent genes that have tandem repeats. (**C**) Hi-C interaction map of *T. versicolor* genome assembly. (**D**) BUSCO assessment of genome completeness and gene completeness for *T. versicolor*.

Sequencing was performed on the PacBio Revio and Illumina HiSeq X high-throughput sequencing platforms, combining long-read and short-read data to achieve high coverage of the genome. Illumina sequencing generated approximately 6.70 Gb of data, with a coverage depth of 143.4×. The average Q30 value was 93.73%, ensuring the genome’s completeness and accuracy (Table S2). K-mer analysis estimated the size of the *T. versicolor* genome to be approximately 48.01 Mb, with a heterozygosity rate of 4.83% (Figure 1A). PacBio sequencing generated 8.51 Gb of long-read data, with an average read length of 18.50 kb (Table S3). Hi-C sequencing generated 48.33 Gb of data, with an average Q30 value of 95.64% (Table S4).

The genome was assembled using PacBio long-read data for redundancy removal and Illumina short-read data for error correction. The final assembled genome size was 47.42 Mb, with an N50 length of 3.79 Mb and a GC content of 57.68%, closely matching the K-mer estimate (Table S5). A genomic circos plot was generated to visually represent the genomic architecture and organization of the 13 high-quality chromosomes assembled in this study (Figure 1B). The plot highlights the distribution of various genomic features, including gene density, intra-species synteny regions, and chromosomal arm length. To investigate the 3D chromosomal interactions, we constructed a Hi-C interaction heatmap that visualizes chromatin interaction frequencies across the 13 chromosomes. Hi-C interaction heat maps reveal strong interactions within chromosomes, indicating the accuracy of chromosome mounting (Figure 1C, Table S6). BUSCO (Benchmarking Universal Single-Copy Orthologs) analysis revealed a genome completeness of 95.80%, demonstrating the integrity and accuracy of the genome assembly (Figure 1D, Table S7).

### 3.2. Genome Annotation

Gene prediction in the *T. versicolor* genome was conducted using both ab initio and homology-based methods. A total of 13,307 protein-coding genes were predicted. The average gene length was 1699.07 bp (Table 1). For the coding sequences (CDS), a total of 72,773 CDS were identified, with an average CDS length of 250.65 bp. In addition, 59,736 introns were identified, with an average intron length of 72.76 bp (Table 1). BUSCO (Benchmarking Universal Single-Copy Orthologs) analysis revealed that 95.90% of the single-copy genes were complete, while 1.9% were partially missing (Figure 1D). Non-coding RNA (ncRNA) analysis predicted a total of 306 ncRNAs in the *T. versicolor* genome, including 277 transfer RNA (tRNA) genes, 21 snRNA genes, 7 snoRNA genes, and 1 small RNA (sRNA) gene (Table S7).

**Figure 2:**
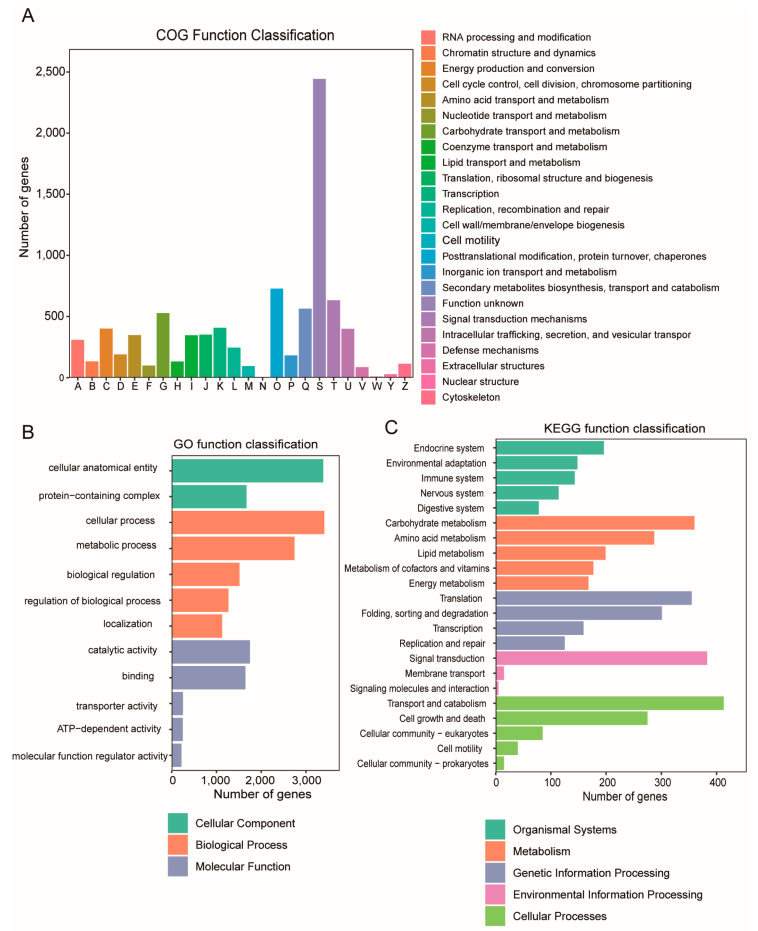
The genomic functional annotation of *T. versicolor* includes classifications based on COG (Clusters of Orthologous Groups of proteins), GO (Gene Ontology), and KEGG (Kyoto Encyclopedia of Genes and Genomes). (**A**) The distribution of *T. versicolor* genes across COG functional categories, comprising 23 major groups. (**B**) The distribution of genes in the GO system, categorized into three main domains: ‘Molecular Function’, ‘Biological Process’, and ‘Cellular Component’. (**C**) The functional distribution of *T. versicolor* genes across KEGG metabolic pathways, grouped into five major domains: ‘Metabolism’, ‘Genetic Information Processing’, ‘Environmental Information Processing’, ‘Cellular Processes’, and ‘Organismal Systems’.

The protein-coding genes were functionally annotated using the NR, Swiss-Prot, KEGG, and GO databases, with 98.67% of the genes successfully annotated (Table S8). In the functional annotation analysis of the *T. versicolor* genome, the COG, GO, and KEGG databases provided comprehensive classifications of functional categories and metabolic pathways. COG annotation identified 124 functional categories, with 464 genes related to secondary metabolites biosynthesis, transport, and catabolism (Figure 2A). GO annotation revealed that a total of 3613 genes were annotated to 9658 GO terms, covering the three main categories: molecular function, cellular component, and biological process (Figure 2B). Genes related to cellular components were primarily associated with ‘protein-containing complex’ and ‘cellular anatomical entity’, while those in molecular function were predominantly linked to ‘catalytic activity’ and ‘binding’. In the biological process category, the main terms included ‘cellular process’ and ‘metabolic process’. KEGG pathway annotation further elucidated the distribution of genes in metabolic networks, successfully mapping 2775 genes to 287 pathways, primarily associated with the ‘endocrine system’, ‘carbohydrate metabolism’, ‘amino acid metabolism’, ‘signal transduction’, and ‘transport and catabolism’ (Figure 2C).

The prediction results for repetitive sequences in the *T. versicolor* genome indicate that approximately 10.04% of the genome consists of repetitive sequences (Table S9). The majority are retrotransposons, accounting for 3.05% of the total genome length, with long terminal repeats (LTRs) and long interspersed nuclear elements (LINEs) being the most abundant types, representing 2.70% and 0.35% of the repetitive sequences, respectively. Among the LTRs, Gypsy/DIRS1 and Ty1/Copia are the two most abundant families, constituting 1.88% and 0.51%, respectively (Table S9). Additionally, DNA transposons and other types of transposons account for 0.90% and 5.51%, respectively. Simple sequence repeats (SSRs) are also distributed throughout the genome, comprising 0.60% of the total genome length (Table S9).

### 3.3. Evolutionary and Comparative Genomic Analysis of T. versicolor

**Figure 3:**
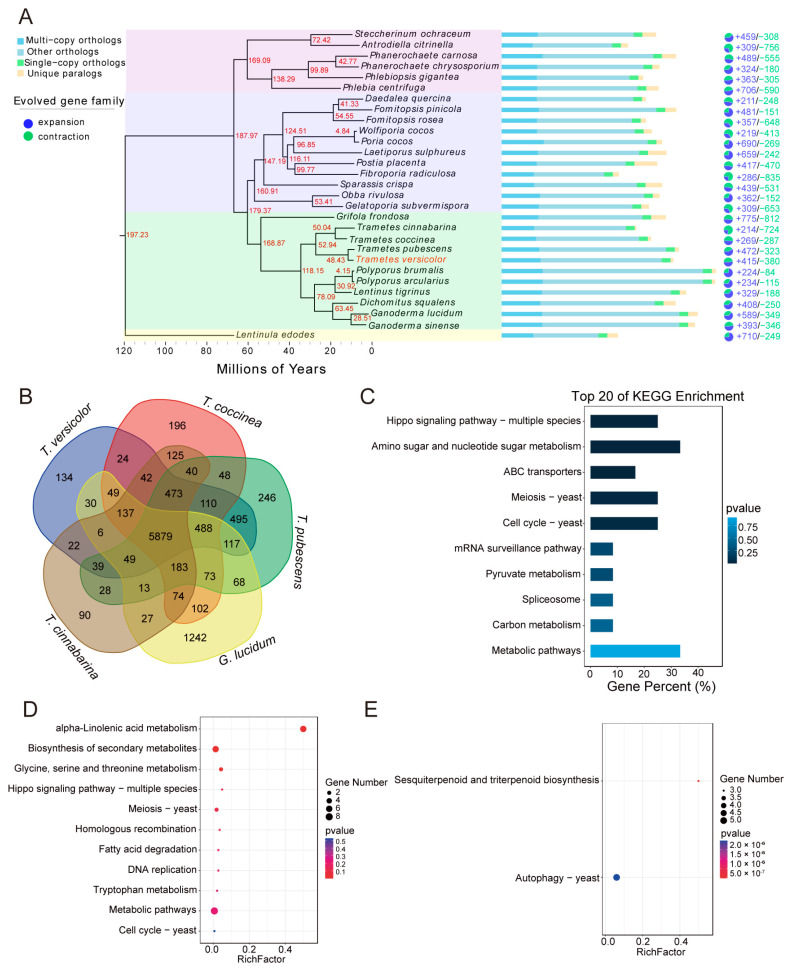
Phylogenetic analysis and gene family evolution of *T. versicolor*. (**A**) A phylogenetic tree constructed using single-copy genes for *T. versicolor* and 28 other fungal species, with *L. edodes* designated as the outgroup. The red numbers indicate divergence times, while the bar chart represents the number of gene family types for each species, including single-copy orthologs, multi-copy orthologs, unique paralogs, and other orthologs. The pie chart shows the numbers of expanded (blue) and contracted (green) gene families. (**B**) The numbers of shared and species-specific gene families between *T. versicolor* and *T. cinnabarina*, *T. coccinea*, *T. pubescens*, and *G. lucidum*. (**C**) KEGG pathway enrichment analysis of gene families specific to *T. versicolor*. (**D**) KEGG functional enrichment analysis of expanded gene families in the *T. versicolor* genome. (**E**) KEGG functional enrichment analysis of contracted gene families in the *T. versicolor* genome.

To investigate the evolutionary relationships between *T. versicolor* and other fungi, a total of 29 species were selected for gene family clustering analysis. Across these 29 species, 383,504 genes were identified, of which 347,860 (90.7%) were assigned to orthogroups. The remaining 35,644 genes (9.3%) were unassigned (Table S10). A total of 20,386 orthogroups were identified, including 4890 species-specific orthogroups comprising 18,359 genes, which account for 4.8% of the total gene set. Among these orthogroups, 2351 contained genes from all species, while 677 were identified as single-copy orthogroups (Figure 3A). To further explore the phylogenetic relationships between *T. versicolor* and other fungi, a phylogenetic tree was constructed using 677 single-copy orthogroups (Figure 3A). The results revealed that *T. pubescens* is the closest relative of *T. versicolor*, forming a distinct clade. This clade is followed by *T. cinnabarina* and *T. coccinea*. Another branch, composed of *G. sinense*, *G. lucidum*, *D. squalens*, *L. tigrinus*, and *P. brumalis*, was identified as the sister clade to the genus *Trametes* (Figure 3A).

To estimate divergence times, molecular clock analysis was performed using fossil calibration points and substitution rates derived from fungal evolutionary studies. The analysis estimated that the genus *Trametes* diverged approximately 118.15 Mya (Figure 3A). The branches of *T. versicolor* and *T. pubescens* form sister branches with the branches of *T. coccinea* and *T. cinnabarina*, and their divergence time was estimated to be approximately 52.94 Mya. The divergence time between *T. versicolor* and *T. pubescens* was estimated to be around 48.43 Mya (Figure 3A).

To further investigate the evolutionary characteristics of gene families in the *T. versicolor* genome, clustering analysis was conducted for *T. versicolor* along with *T. cinnabarina*, *T. coccinea*, *T. pubescens*, and *G. lucidum* (Figure 3B). The analysis revealed 5879 shared gene families among these species. Compared to the other four species, *T. versicolor* contains 134 unique gene families comprising 290 genes (Figure 3B). KEGG annotation of these unique genes showed significant enrichment in pathways such as ‘metabolic pathways’, ‘amino sugar and nucleotide sugar metabolism’, and ‘ABC transporters’ (Figure 3C). Additionally, an analysis of gene family contraction and expansion based on the phylogenetic tree identified 415 expanded and 280 contracted gene families in *T. versicolor* (Figure 3A). The expanded gene families were significantly enriched in KEGG pathways related to ‘alpha-linolenic acid metabolism’, ‘biosynthesis of secondary metabolites’, and ‘glycine, serine, and threonine metabolism’ (Figure 3D). Conversely, the contracted gene families were predominantly enriched in pathways such as ‘sesquiterpenoid and triterpenoid biosynthesis’ and ‘autophagy–yeast’ (Figure 3E).

### 3.4. Comparative Genomic Analysis of T. versicolor

**Figure 4:**
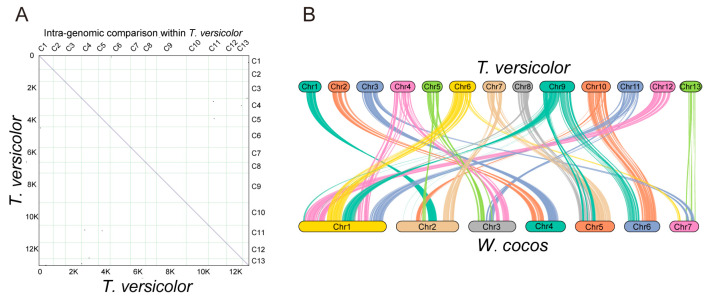
Comparative genomics analysis of intra-species and inter-species. (**A**) Self-synteny analysis of the *T. versicolor* genome. (**B**) Gene-based synteny analysis of *T. versicolor* and *W. cocos* chromosomes.

To investigate the synteny within the *T. versicolor* genome, we performed a self-synteny analysis. A total of 21 synteny blocks were identified, containing 284 genes (Figure 4A). Additionally, we identified 8586 conserved genomic regions between the two species and visualized the gene positions and arrangements within these regions. Further comparative analysis was performed between *W. cocos* and *T. versicolor* genomes. In the comparative genomics analysis, we identified 366 synteny blocks between the two species (Figure 4B). Among these, chromosome 13 in *T. versicolor* and chromosome 7 in *W. cocos* exhibit significant gene arrangement consistency, indicating that these regions have been conserved during evolution. These syntenic regions covered 34.42% (8586) of the genes in the *T. versicolor* and *W. cocos* genomes, involving all chromosomes (Figure 4B).

### 3.5. Effects of Light and Dark Conditions on the Growth, Dry Weight, and Polyphenol and Triterpene Content of T. versicolor

**Figure 5:**
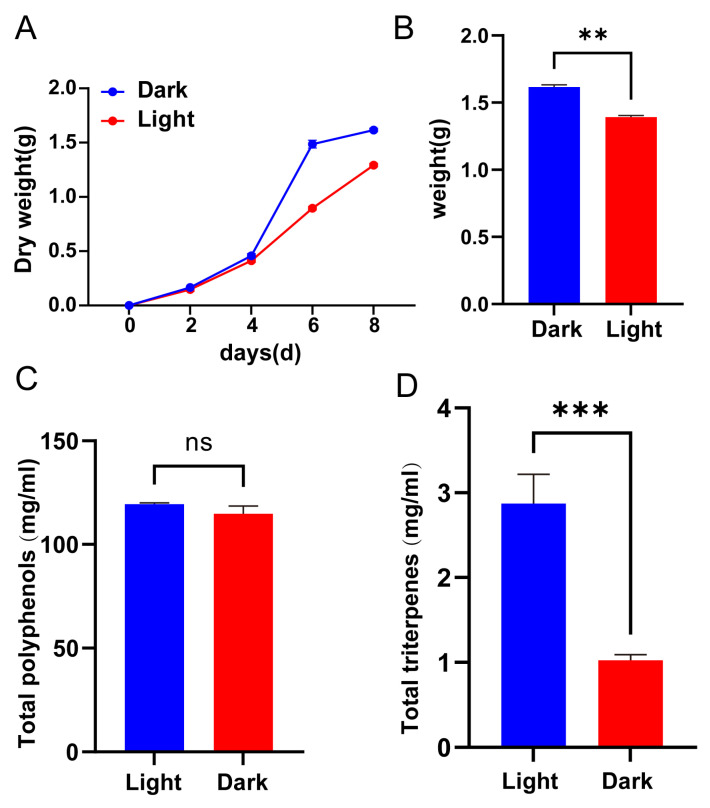
The differences in growth dynamics and metabolite accumulation of *T. versicolor* under light and dark conditions. (**A**) Dry weight dynamics of *T. versicolor* over time under light and dark conditions. (**B**) Final dry weight of *T. versicolor* under light and dark conditions. (**C**) Total polyphenol content of *T. versicolor* under light and dark conditions. (**D**) Total triterpene content of *T. versicolor* under light and dark conditions. Error bars represent the mean ± standard deviation (SD) of three biological replicates. Statistical significance was determined using Student’s *t*-test: ns indicates no significant difference; ** *p* < 0.01; *** *p* < 0.001.

To analyze the growth and metabolic changes of *T. versicolor* under normal dark conditions and light stress treatments, the trends in dry weight over time, as well as the dry weight, polyphenol content, and total triterpene content, were compared across different light environments. Under dark conditions, the dry weight of *T. versicolor* increased proportionally with time (Figure 5A). However, under light stress conditions, the dry weight accumulation was lower compared to that in the dark. Although the dry weight of the fungus showed a gradual increase over time under light stress, the growth rate was significantly slower than that under dark conditions. Measurements of total dry weight revealed that the final weight of *T. versicolor* under dark conditions was significantly higher than that under light stress treatment (*p* < 0.01) (Figure 5B). These findings suggest that light exposure not only reduces the growth rate of *T. versicolor* but also limits the overall biomass accumulation. The total polyphenol content under light stress was slightly higher than that under dark conditions, but the difference was not statistically significant (ns) (Figure 5C). In contrast, the total triterpene content was significantly higher under light stress compared to dark conditions (*p* < 0.001) (Figure 5D), indicating that light stress might promote the synthesis of triterpenoid compounds through the regulation of secondary metabolic pathways.

### 3.6. Characteristics of CAZymes and P450s in the T. versicolor Genome

**Figure 6:**
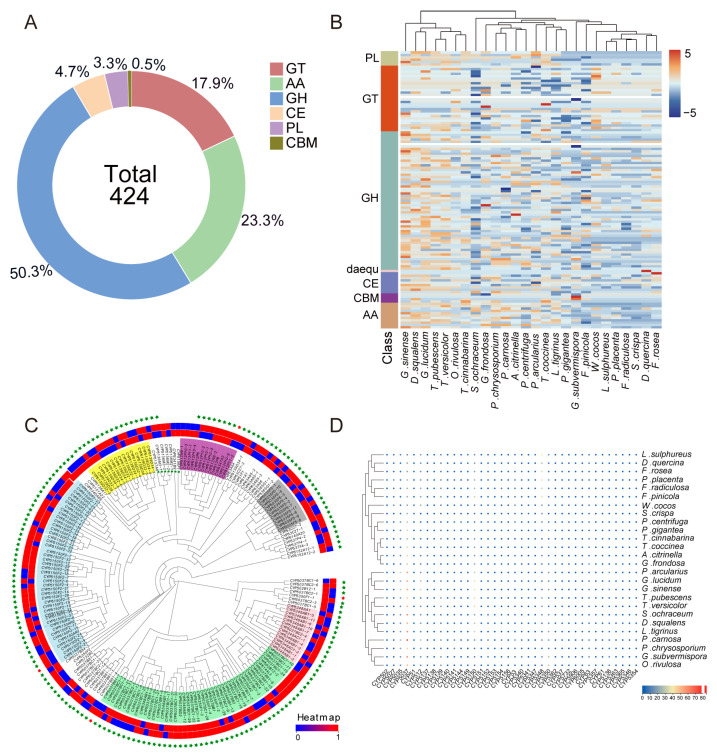
The identification and classification of CAZymes and CYP450 enzymes in the genome of *T. versicolor*. (**A**) The total number and proportional distribution of CAZymes in the *T. versicolor* genome. (**B**) A comparative analysis of CAZyme family components between *T. versicolor* and other fungi. (**C**) The phylogenetic analysis of CYP450 enzymes in *T. versicolor*. Light blue: CYP5150, light green: CYP5037, pink: CYP5348, yellow: CYP5139, purple: CYP512, gray: CYP5035. From inside to outside, the color changes from red to blue, indicating that the expression in dark is higher than in light; from blue to red, indicating that the expression in light is higher than in dark. Two red colors indicate no significant difference, and the outer green stars represent non-differential genes, while red stars represent differential genes. (**D**) The quantity and clustering analysis of CYP450 enzymes in *T. versicolor* and other species.

CAZymes and P450s play key roles in the secondary metabolism of fungi. To investigate the characteristics of the CAZyme and CYP450 families in the *T. versicolor* genome, both enzymes were identified and compared across *T. versicolor* and other species. A total of 424 CAZymes were identified in the *T. versicolor* genome, which were classified into six major families: glycoside hydrolases (GH, 50.3%), auxiliary activities (AA, 23.3%), glycosyltransferases (GT, 17.9%), carbohydrate esterases (CE, 4.7%), polysaccharide lyases (PL, 3.3%), and carbohydrate-binding modules (CBM, 0.5%) (Figure 6A). Among these families, GH and AA are the major functional families. Comparative analysis showed that in 26 fungal species, the GH and GT families had the highest proportions, while CBM had the smallest proportion (Figure 6B). Compared to other fungi, *T. versicolor* has a significantly higher number of auxiliary activity enzymes (AA), while the number of glycoside hydrolases (GH) is comparable to other species.

A total of 192 CYP450 genes were identified in the *T. versicolor* genome. Phylogenetic analysis revealed that the CYP450 enzymes in *T. versicolor* were divided into six subfamilies: CYP5150, CYP5037, CYP5348, CYP5139, CYP512, and CYP5035. Among these subfamilies, CYP5037 and CYP5150 were the two most abundant subfamilies (Figure 6C). Cluster analysis with other fungi showed that the abundance of CYP5037 in *T. versicolor* was significantly higher than that of other subfamilies. Phylogenetic relationships and the number of P450 genes indicate that closely related species have similar quantities (Figure 6D). For example, compared to other fungi, *T. versicolor*, along with *T. pubescens*, *S. ochraceum*, *D. squalens*, *L. tigrinus*, *G. sinense*, *G. lucidum*, and *P. arcularius*, showed more conserved numbers of CYP5037, CYP5150, and other genes.

### 3.7. Transcriptome Analysis Revealed Changes in Gene Expression in the Mycelium of T. versicolor Under Light Treatment Conditions

**Figure 7:**
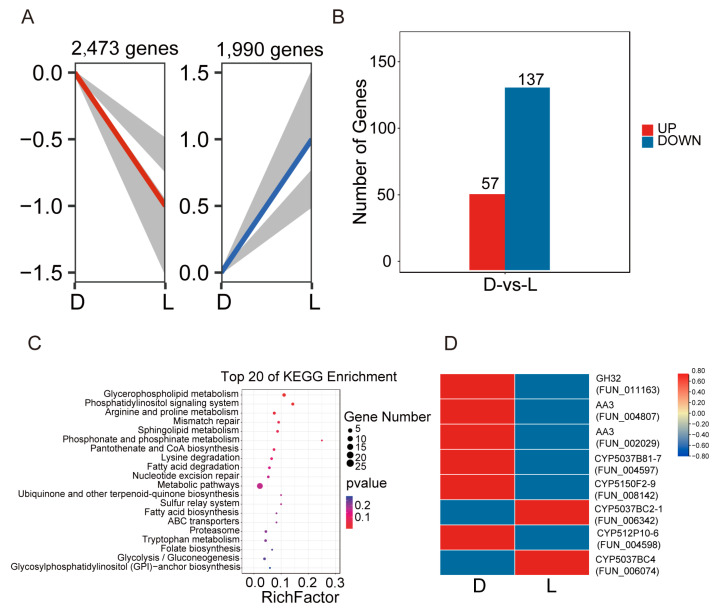
Transcriptome analysis of *T. versicolor* mycelium under dark and light conditions. (**A**) Expression trend analysis of all genes. (**B**) Number of differentially expressed genes (upregulated and downregulated) under dark and light stress treatment. (**C**) KEGG enrichment analysis of the top 20 pathways for differential genes. (**D**) Heatmap of the expression levels of significantly changed CAZymes and P450s genes under dark and light conditions, with colors ranging from red to blue to indicate upregulation and downregulation.

To investigate the effects of light treatment on the expression levels of key genes in the mycelium of *T. versicolor*, transcriptome analyses were conducted under dark conditions (control group) and light conditions (treatment group). A total of 30.08 Gb of transcriptomic sequencing data was obtained, with an average Q30 of 90.23% (Table S11). To analyze the changes in gene expression under light stress, differentially expressed genes were identified. Trend analysis revealed significant dynamic changes in gene expression under light treatment, with 2473 genes showing upregulation and 1990 genes showing downregulation (Figure 7A). Comparison between dark and light conditions revealed 194 differentially expressed genes in *T. versicolor*, including 57 upregulated genes and 137 downregulated genes, indicating that light significantly influences gene expression (Figure 7B). KEGG enrichment analysis of the differentially expressed genes showed significant enrichment in the pathways of ‘glycerophospholipid metabolism’, ‘ubiquinone and other terpenoid-quinone biosynthesis’, and ‘metabolic pathways’, suggesting that these pathways may be involved in light response regulation (Figure 7C). Analysis of the significantly changing CAZymes genes in *T. versicolor* revealed that two AA3 genes (*FUN_004807*, *FUN_002029*), and one GH gene (*FUN_011163*) exhibited significant downregulation. Under light conditions, the expression levels of several P450s genes in *T. versicolor* changed significantly. For example, compared to dark conditions, the expression levels of *CYP5037B81-7* (*FUN_004597*), *CYP5150F2-9* (*FUN_008142*), and *CYP512P10-6* (*FUN_004598*) were significantly upregulated under light conditions, while the expression levels of *CYP5037BC2-1* (*FUN_006342*) and *CYP5037BC4* (*FUN_006074*) were significantly downregulated (Figure 7D).

### 3.8. qRT-PCR Analysis

**Figure 8:**
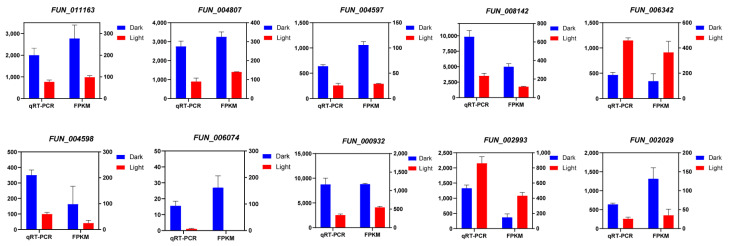
Validation of RNA-seq data using qRT-PCR. Relative expression levels of 10 selected genes (*FUN_011163*, *FUN_004807*, *FUN_002029*, *FUN_004597*, *FUN_008142*, *FUN_006342*, *FUN_004598*, *FUN_006074*, *FUN_000932*, and *FUN_002993*) as determined by qRT-PCR. Bars represent the mean ± standard deviation of three biological replicates. The bar graph shows gene expression levels under different treatments.

To confirm the accuracy of the transcriptome data, we performed quantitative real-time PCR (qRT-PCR) on 10 randomly selected genes, including Cazymes and P450 genes (*FUN_011163*, *FUN_004807*, *FUN_002029*, *FUN_004597*, *FUN_008142*, *FUN_006342*, *FUN_004598*, *FUN_006074*) (Figure 8). The results showed that the expression patterns observed through qRT-PCR were highly consistent with the RNA-seq data, demonstrating the reliability and reproducibility of the transcriptomic analysis (Figure 8). Minor differences in expression levels between the two methods were attributed to differences in sensitivity and detection platforms.

### 3.9. Transporter Proteins, Membrane Proteins, and Secondary Metabolite Clusters in the T. versicolor Genome

**Figure 9:**
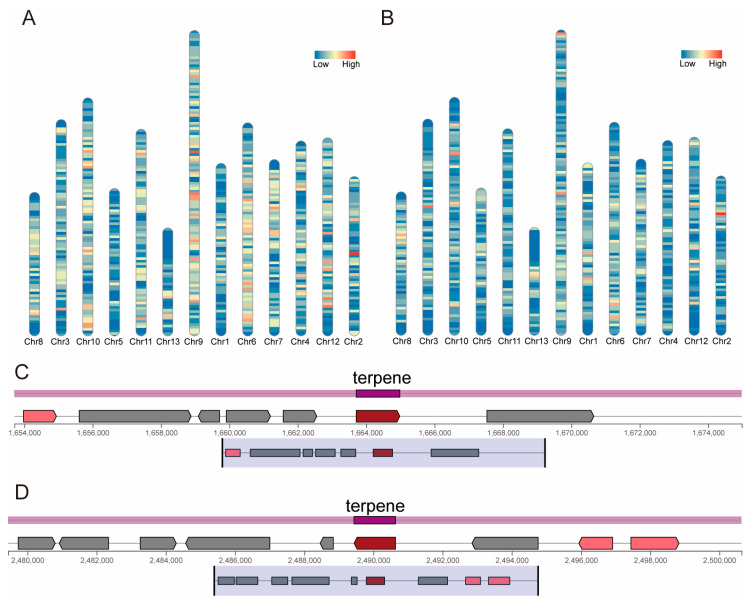
Transporter proteins, membrane proteins, and secondary metabolite clusters in the *T. versicolor* genome. (**A**) Distribution of transporter proteins across the chromosomes of the *T. versicolor* genome. (**B**) Distribution of membrane proteins across the chromosomes of the *T. versicolor* genome. (**C**) A gene cluster associated with light-sensitive triterpene metabolism located on chromosome 12 (1,653,710–1,674,983). (**D**) A gene cluster associated with light-sensitive triterpene metabolism located on chromosome 12 (2,479,443–2,500,639).

To investigate the function and mechanisms of transporter proteins, we identified the distribution of transporter proteins and membrane proteins in the *T. versicolor* genome. A total of 2577 transporter proteins were identified, with the largest proportion being electrochemical potential-driven transporters, accounting for 23.1% of the total (Table S12). This was followed by accessory factors involved in transport and channels/pores, which accounted for 21.9% and 21.8%, respectively. The transporter proteins identified in the *T. versicolor* genome were mapped to the chromosomes, revealing their distribution across all chromosomes, with numbers ranging from 72 to 321 per chromosome (Figure 9A). Chromosome 9 contained the highest number, with 321 transporter proteins, while chromosome 13 had the fewest, with 72 transporter proteins. Further analysis identified 2582 membrane proteins, which were distributed across all chromosomes, with numbers ranging from 67 to 421 (Figure 9B). Chromosome 9, again, had the highest number, with 421 membrane proteins, while chromosome 13 had the fewest, with 67.

To better understand the metabolic products and their biological functions, we also identified secondary metabolite gene clusters in the *T. versicolor* genome. A total of 33 secondary metabolite clusters were identified, including 17 triterpene biosynthesis clusters, 9 NRPS clusters, 5 RIPP clusters, and 2 T1PKS clusters (Table S13). Among the 17 triterpene clusters, 2 were identified as light-sensitive, potentially linking light exposure to triterpene synthesis in *T. versicolor*. These clusters are located on chromosome 12 at positions 1,653,710–1,674,983 (Figure 9C) and 2,479,443–2,500,639 (Figure 9D).

## 4. Discussion

In this study, the genome of *Trametes versicolor* was sequenced, assembled, and annotated with high quality using a combined strategy of PacBio Revio long-read sequencing and Illumina HiSeq X short-read sequencing. This approach, integrating both long- and short-read data, achieved high genome coverage and accuracy. Building upon the contig-level assembly, the application of Hi-C technology further generated a high-quality chromosome-level genome. Through these techniques, we successfully obtained a 47.42 Mb high-quality genome sequence, and BUSCO analysis confirmed a genome completeness of 95.80%, indicating that our genome assembly reached a high level of quality, providing a reliable foundation for subsequent functional analysis and research.

Compared to other published fungal genomes, the *T. versicolor* genome demonstrates significant advantages in chromosome-level assembly, gene prediction, and functional annotation. Specifically, the high-quality assembly of 13 chromosomes provides a valuable reference for genomic research, with a genome size of 47.42 Mb and a scaffold N50 of 3.79 Mb, surpassing the NCBI-released v1.0 (https://www.ncbi.nlm.nih.gov/datasets/genome/GCF_000271585.1/, accessed on 16 October 2024), which had a scaffold N50 of 2.9 Mb. This chromosome-level assembly enhances the accuracy of genome annotation, facilitating genomic evolution studies and the potential for genomic manipulation. A total of 13,307 protein-coding genes were identified, with a successful annotation rate of 98.67%, showing slight differences from the 14,562 genes identified in the v1.0 version. Overall, this study significantly improves assembly quality and annotation accuracy, providing a high-quality chromosome-level genome that serves as a powerful resource for *T. versicolor* ecological research, functional gene discovery, and industrial applications, offering valuable insights for the study of other wood-degrading fungi.

The evolutionary analysis of the *T. versicolor* genome, based on gene family clustering with 29 fungal species, identified a unique branch in the *Trametes* genus, closely related to *T. pubescens*, *T. cinnabarina*, and *T. coccinea*. Phylogenetic tree construction revealed that *T. versicolor* is most closely related to *T. pubescens*, forming an independent clade and confirming its unique position within the *Trametes* genus. This evolutionary relationship provides background information for further functional studies of the *T. versicolor* genome and important insights into the evolutionary process of wood-decaying fungi. The divergence time analysis, based on molecular clock analysis, estimated that the *Trametes* genus diverged approximately 118.15 million years ago, and the divergence between *T. versicolor and T. pubescens* occurred around 48.43 million years ago, indicating a considerable evolutionary distance between the species. This result aligns with existing fungal evolutionary studies and further supports the unique ecological role of *T. versicolor* in wood-decaying fungi [57]. Through gene family clustering analysis, we also found that *T. versicolor* has 134 species-specific gene families, associated with key biological pathways such as ‘metabolism’, ‘amino sugar and nucleotide sugar metabolism’, and ‘ABC transporters’. These findings emphasize the adaptability of *T. versicolor* in terms of its metabolic and transport mechanisms [58]. Gene family expansion was closely linked to secondary metabolite synthesis, particularly in pathways such as ‘secondary metabolite biosynthesis’, suggesting that these expanded genes may play a role in the production of *T. versicolor*’s unique bioactive compounds. Conversely, gene family contraction primarily occurred in pathways related to ‘sesquiterpenoid and triterpenoid biosynthesis’ and ‘autophagy–yeast’, indicating that *T. versicolor* may use autophagy mechanisms to cope with environmental stress and enhance adaptability [59]. Overall, the expansion and contraction of gene families in the *T. versicolor* genome enhance its ability to adapt to diverse environments, particularly in secondary metabolism and environmental response mechanisms, providing important insights for its biological properties and potential applications.

In fungal secondary metabolism, carbohydrate-active enzymes (CAZymes) and CYP450 enzymes play crucial roles in the degradation and metabolism of carbohydrates, as well as the regulation of drug synthesis and breakdown [60,61]. By identifying and comparing the CAZyme and CYP450 families in *T. versicolor* and other fungal species, this study provides valuable genomic insights into the metabolic mechanisms of *T. versicolor* and its adaptability to different environments. The CAZyme analysis in the *T. versicolor* genome reveals its central role in carbohydrate degradation and conversion. Specifically, glycoside hydrolases (GH) and auxiliary activity enzymes (AA) are the most abundant enzyme families, accounting for 50.3% and 23.3% of the total enzymes, respectively. This result, consistent with other fungal species, highlights the importance of GH and AA families in fungal carbohydrate metabolism [62,63,64,65]. GH enzymes are key for breaking down complex polysaccharides, playing a crucial role in the degradation of wood and plant matter by wood-decaying fungi, while AA enzymes are involved in the breakdown of lignocellulose, likely contributing to the growth and nutrient acquisition of *T. versicolor*. Notably, *T. versicolor* has a significantly higher number of AA family members compared to other fungi, suggesting a strong adaptation in utilizing various carbon sources and in biodegradation processes. This finding aligns with previous research, indicating that auxiliary activity enzymes play a vital role in fungi’s ability to adapt to different environments by enhancing carbon source utilization.

Regarding the CYP450 enzyme family, we identified 192 CYP450 genes in *T. versicolor*, which were classified into six subfamilies through phylogenetic analysis [66,67]. Notably, CYP5037 and CYP5150 are the most abundant subfamilies in the *T. versicolor* genome, suggesting their core role in biosynthesis and metabolic pathways. CYP450 enzymes in fungi are involved not only in drug metabolism but also in the synthesis of secondary metabolites, such as antibiotics, toxins, and other bioactive compounds. Compared to other fungi, *T. versicolor* has a higher abundance of CYP5037 and CYP5150 subfamilies, further supporting the importance of these genes in the synthesis of the unique metabolites of *T. versicolor*. This is particularly evident when compared with *T. pubescens*, *S. ochraceum*, *D. squalens*, and other species, where *T. versicolor* exhibits a richer presence of these key genes, indicating its potentially unique metabolic functions and ecological adaptation mechanisms [68]. The conservation of the CYP450 gene family also provides insights into fungal evolution [69]. Gene comparison with other species reveals a high degree of gene conservation between *T. versicolor* and closely related species, especially in the abundance of CYP5037 and CYP5150. This suggests that these genes may play a universal role in fungal metabolic regulation and environmental adaptation. Further functional studies are needed to explore the specific roles of these expanded CYP450 genes in the secondary metabolism of *T. versicolor*, particularly in stress resistance and biodegradation processes. Overall, the study of the CAZyme and CYP450 families reveals the significant roles of *T. versicolor* in carbohydrate degradation, metabolic regulation, and secondary metabolite synthesis.

In this study, we compared the transcriptomic data of *T. versicolor* under dark and light conditions, revealing the significant impact of light treatment on gene expression in the mycelium. Previous research has demonstrated that light is an essential environmental signal regulating fungal physiology and metabolism [70,71,72]. It acts through photoreceptor systems to activate signaling pathways, thereby modulating gene expression. The differentially expressed genes (DEGs) and enriched metabolic pathways identified in this study further support this notion, indicating that light signals can trigger a complex network of gene regulatory mechanisms. KEGG enrichment analysis revealed significant enrichment in pathways such as ‘glycerophospholipid metabolism’, ‘ubiquinone and other terpenoid-quinone biosynthesis’, and ‘metabolic pathways’. This suggests that light signals may regulate these metabolic pathways to modulate membrane fluidity, stability, and energy metabolism, aiding *T. versicolor* in adapting to light environments. These findings align with previous studies showing that light influences fungal growth and metabolism, for instance, by regulating lipid metabolism to affect fungal morphology and secondary metabolite production. Moreover, the significantly downregulated CAZymes genes (such as AA3 and GH family genes) and the substantial changes in P450 genes provide further insights into the regulatory effects of light treatment on key metabolic enzymes and detoxification systems [73]. As a crucial family of monooxygenases, the expression changes in P450 genes suggest that light signals may influence the secondary metabolism and xenobiotic degradation capacity of *T. versicolor*. For example, the significant upregulation of CYP5037 and CYP5150 genes indicates their potential roles in light-responsive regulation, while the downregulation of other genes might serve as a protective mechanism to adapt to light conditions. This dynamic pattern of gene expression likely reflects a differentiated metabolic strategy of fungi under light exposure. In conclusion, this study offers new insights into the light response mechanisms of *T. versicolor* through an in-depth analysis of DEGs and their associated pathways. Future research could further validate the specific roles of these genes and pathways in light responses and explore the effects of light treatment on the production and diversity of secondary metabolites, providing a theoretical foundation for the industrial application of light in regulating fungal metabolism.

In this research, we found that light significantly affects triterpene biosynthesis in *T. versicolor*. As a typical saprophytic fungus, *T. versicolor* produces secondary metabolites, especially triterpenes, which possess important biological activities, such as antimicrobial and antioxidant properties [74,75]. By analyzing the genome and transcriptome data, we identified gene clusters related to triterpene biosynthesis in *T. versicolor*, and further discovered two light-sensitive triterpene biosynthesis clusters. This suggests that light may regulate the expression of these gene clusters, thereby affecting triterpene synthesis. Light, as an environmental factor, has been widely studied for its role in secondary metabolism in plants and other fungi [76]. It can activate or suppress the expression of genes involved in metabolic pathways by regulating specific transcription factors or signaling pathways [72]. In *T. versicolor*, the discovery of light-sensitive triterpene gene clusters further supports this hypothesis, indicating that light may regulate the activity of these clusters, promoting or inhibiting triterpene synthesis. The influence of light on secondary metabolites may occur by altering the concentration of certain key metabolites within the cell, thereby regulating metabolic flux and product synthesis [77]. It is noteworthy that different wavelengths of light may have varying effects on triterpene synthesis in *T. versicolor*, possibly related to specific regulatory mechanisms within the light-sensitive gene clusters. Therefore, further research on the effects of light intensity, spectrum, and exposure time on triterpene biosynthesis will help deepen our understanding of light’s regulatory role in the secondary metabolism of *T. versicolor* and provide a theoretical basis for the efficient production of triterpene-based natural products.

## 5. Conclusions

This study completed the whole-genome sequencing, assembly, and annotation of *T. versicolor*, constructing a high-quality reference genome that lays a solid foundation for in-depth research into its genetic characteristics and biological functions. Through gene family clustering analysis, we systematically investigated the unique gene families of *T. versicolor* and the dynamics of gene family expansion and contraction, identifying key gene groups associated with its ecological adaptability and metabolic functions. Additionally, we identified the CYP450 and CAZyme families in the genome of *T. versicolor*, providing deeper insights into their potential roles in metabolic regulation. Transcriptome data were utilized to analyze the gene expression patterns of *T. versicolor* under dark and light conditions. The results demonstrated that light significantly affects the expression of certain genes in *T. versicolor*. The identification of light-sensitive triterpene biosynthesis clusters suggests that environmental factors like light may influence secondary metabolite production, offering new insights into the regulation of triterpene synthesis in *T. versicolor*. This study systematically elucidates the genomic structure, functional gene families, and light-responsive regulatory mechanisms of *T. versicolor*, providing a theoretical foundations and data support for further exploration of its metabolic potential, optimization of its industrial applications, and understanding of the ecological adaptability of white-rot fungi.

## Figures and Tables

**Table 1:** Statistics on gene structure annotation in *T. versicolor* genome.

Characteristics	Value
Number of genes	13,307
Average gene length	1699.07
Average gene cds length	1370.75
Total number of cds	72,773
Total cds length	18,240,611
Average cds length	250.65
Average number of cds per gene	5.47
Total number of exons	72,773
Total exon length	18,240,611
Average exon length	250.65
Average number of exons per gene	5.47
Number of genes with introns	11,869
Total number of introns	59,736
Total intron length	4,346,185
Average intron length	72.76
Average number of introns per gene	4.49

## Data Availability

The genome of *T. versicolor* project has been submitted to GenBank SRA (PRJNA1195250). RNA-seq data have been submitted to GeneBank SRA (PRJNA1195334).

## Supplementary Materials

The following supporting information can be downloaded at: https://www.mdpi.com/article/10.3390/jof11010081/s1, Table S1. qRT-PCR primers sequence; Table S2: the Illumina short reads of *T. versicolor*; Table S3: The HiFi reads of *T. versicolor*; Table S4: The Hi-C data of *T. versicolor*; Table S5: The final assembled genome of *T. versicolor*; Table S6: The final chromosome-level assembly genome of *T. versicolor*; Table S7: Statistics on small RNAs in *T. versicolor* genome; Table S8: Statistics on gene function annotation in *T. versicolor* genome; Table S9: Classification of repetitive elements in the genome of *T. versicolor*; Table S10: Cluster analysis of 29 fungi gene families based on genomic data; Table S11: Transcriptome sequencing data of mycelium of *T. versicolor* subjected to 5-day treatment under normal light and dark stress conditions. Table S12: Classification of transporter proteins in the *T. versicolor* genome; Table S13: Classification of secondary metabolic clusters in the *T. versicolor* genome.
